# Assembly, annotation, and analysis of the first complete chloroplast genome of medicinal plant *Pentas lanceolata* (Forssk.) Deflers

**DOI:** 10.1080/23802359.2026.2753257

**Published:** 2026-11-06

**Authors:** Xiujuan Yin, Rui Xu, Chunfeng Wan, Junliu Song, Jintong Wang, Liqiang Wang

**Affiliations:** a College of Pharmacy, Heze University, Heze, PR China; b College of Agriculture and Bioengineering, Heze University, Heze, PR China

**Keywords:** *Pentas lanceolata*, complete chloroplast genome, phylogenetic analysis, next generation sequencing

## Abstract

*Pentas lanceolata* (Rubiaceae) is a perennial herb with ornamental and medicinal value, but lacks chloroplast (cp) genomic resources. Herein, we report its first complete cp genome: a 154,476 bp circular molecule (37.82% GC) with typical quadripartite structure, including LSC (84,407 bp, 35.79% GC), SSC (17,175 bp, 32.21% GC), and two IRs (26,447 bp each, 42.89% GC). Annotation recovered 127 genes (107 unique): 82 protein-coding (76 unique), 37 tRNA (27 unique), and eight rRNA (four unique) genes. IR boundaries show contraction and expansion. Phylogenetic analysis placed it in a clade associated with tribe Spermacoceae (bootstrap = 100%). These findings support evolutionary and genetic improvement studies of *P. lanceolata*.

## Introduction

*Pentas lanceolata* (Forssk) Deflers 1889 is a perennial herb of the Rubiaceae family, increasingly valued for its dual ornamental and medicinal properties (Adhikary 2020). Modern studies have demonstrated its diverse bioactivities, including antiplasmodial (Kigondu et al. 2011; Endale et al. 2012), analgesic (Suman et al. 2014), antinociceptive (Nyangacha et al. 2016), immunomodulatory effects (Abd-Alla et al. 2017), and anti-inflammatory (Kura et al. 2018), highlighting its potential as a source of therapeutic agents.

Rubiaceae ranks among the largest angiosperm families, with over 14,000 species in ∼580 accepted genera and complex taxonomy (Razafimandimbison and Rydin 2024a, 2024b). Although *P. lanceolata*, a Rubiaceous species, has promising medicinal potential backed by phytochemical studies (Venditti et al. 2015; Fahmy et al. 2020; Afifi et al. 2024), its intrafamilial taxonomic position remains unresolved.

Chloroplast (cp) genomes are powerful tools for plant evolutionary and germplasm research owing to their conserved structure, maternal inheritance, and strong phylogenetic signals (Shi et al. 2026). However, genomic studies of the genus *Pentas* remain limited, and no complete cp genome of *P. lanceolata* has been reported, though a partial *P. lanceolata* cp genome (KY378685.1) is deposited in GenBank. We assembled and characterized its first complete cp genome using next‑generation sequencing and resolved its placement within Rubiaceae by maximum‑likelihood (ML) analysis of concatenated shared single‑copy protein‑coding genes (PCGs). This cp genome can provide a valuable genomic resource for future molecular identification, comparative genomics, phylogenetics, and marker-development studies in this medicinally and economically important genus.

## Materials and methods

Samples of *P. lanceolata* were collected and identified by Chunfeng Wan and Junliu Song in October 2025, from Mudan District, Heze City, Shandong Province, China (35.271538° N, 115.460440° E) (Figure 1). A voucher specimen (HZU-2025-1001) was deposited in the Herbarium of Heze University (www.hezeu.edu.cn, contact: Junliu Song, 1467673393@qq.com).

Total genomic DNA was extracted with a Plant Genomic DNA Kit and fragmented into 200–400 bp for building 150 bp paired-end libraries. Libraries were sequenced on the Illumina NovaSeq 6000 platform (San Diego, CA). Raw reads were quality-assessed by FastQC (v0.12.1) and trimmed via Trimmomatic v0.39 (parameters: ILLUMINACLIP:adapter.fa:2:30:10 LEADING:3 TRAILING:3 SLIDINGWINDOW:4:15 MINLEN:36) (Bolger et al. 2014). Clean reads were assembled using GetOrganelle (v1.7.6.1) (Jin et al. 2020; -R 100 -k 21,45,65,85,105 -F embplant_pt -t 30). Initial annotation was performed in CPGAVAS2 (Shi et al. 2019), followed by manual curation in Apollo (v1.11.6) (Firtina et al. 2020) to verify start/stop codons, *rps*12 trans‑splicing, and LSC/IR/SSC junctions. Sequencing depth and coverage map were calculated using the DrawSeqDepth. CPGview (Liu et al. 2023) was utilized to exhibit the cp genome map and the cis-/trans-splicing gene structures. SSRs and long repetitive sequences (LRSs) were statistically analyzed using CPStools (v3.0) (Huang et al. 2024). Software and machine details are provided in Appendix I.

To resolve the phylogenetic placement of *P. lanceolata*, over 54 cp genomes were downloaded from published resources (Yik et al. 2021; Shi et al. 2024; Kang et al. 2026) and screened in Gepard (v1.40) (Krumsiek et al. 2007) to exclude sequences with missing or ambiguous regions. Fifty-four cp genomes shared single-copy PCGs were obtained from 51 Rubiaceae ingroups (including *P. lanceolata*) and three Actinidiaceae outgroups. MAFFT (Katoh and Standley 2016) was used for sequence alignment; aligned sequences were concatenated into a 38,806-site matrix with no trimming or partitioning. ML tree reconstruction was performed in IQ-TREE (v2.0) (Nguyen et al. 2015; Minh et al. 2020; command: ‘iqtree -s concatenation.fas -m MFP -B 1000 -bnni’). ModelFinder identified TVM + F + I + R3 as the optimal model under BIC, and ultrafast bootstrapping (1000 replicates, ‘-B’) assessed branch support. The tree was visualized in FigTree (v1.4.5).

## Results

From 36,884,085 raw 150-bp paired reads, 36,389,201 (98.66% of raw reads) clean paired reads were obtained. A total of 2,114,804 were cp-derived (∼5.73% of raw reads) and used to assembled the cp genome, with minimum, maximum, and average coverage depths of 1125×, 6145×, and 3976.31×, respectively (Figure S1). The cp genome was 154,476 bp (LSC: 84,407 bp; SSC: 17,175 bp; IRs: 26,447 bp each) (Figure 1) with overall GC content 37.82%. GC values for LSC, SSC, and IR were 35.79%, 32.21%, and 42.89%, respectively, with IR region showing higher GC content (Table S1).

Annotation recovered 127 genes (107 unique): 82 protein‑coding (76 unique), eight rRNA (four unique), and 37 tRNA genes (27 unique) (Figure 2, Table S2). Twenty genes exhibit multiple copies: 17 genes from IR duplication (six PCGs, seven tRNA, four rRNA), and three from LSC-located *trnM* (×2) and *trnE* (×3) duplications. Sixteen PCGs have one intron, while *clpP* and *ycf3* each contain two introns (Figure S2, Table S3). The trans‑spliced *rps*12 has its 3′ end in the IR region, with an intron in each 3′ copy (Figure S3). All PCGs encode 26,188 codons; third-position A/U dominates (69.22%), reflecting typical A/U bias. Terminal base frequencies are 31.54% (A), 37.68% (U), 16.49% (G), and 14.28% (C) (Table S1).

Repeat analysis identified 20 SSRs (predominantly A/T‑rich and mainly distributed in intergenic spacers) and 20 LRSs screened with ≥30 bp length, ≤three Hamming distance and ≥90% identity, which were mostly located in intergenic regions (Figure S4). Four IR/SC junctions from 10 cp genomes were characterized (Figure S5). The *rpl2* lies within IR regions across all taxa except *Actinidia chinensis* and *Leptodermis kumaonensis*. In *P. lanceolata*, the *rps19* is slightly farther from the JLA (Figure S5) boundary than other genomes, revealing slight structural variation. The *ycf1* exhibits substantial positional variation near the JSA boundary with no conserved pattern.

In the concatenated PCG tree, *P. lanceolata* was recovered as a monophyletic lineage sister to the tribe Spermacoceae (bootstrap = 100%). However, Spermacoceae exhibited internal heterogeneity, containing several distantly related members (Figure 3).

## Discussion and conclusions

In this study, we report the first complete cp genome of *P. lanceolata* (154,476 bp, 37.82% GC), harboring 127 predicted genes. IR boundary analysis revealed contraction and expansion events, and phylogenetics confirmed its close affinity to tribe Spermacoceae. This genomic resource supports evolutionary and taxonomic investigations of this species.

The cp genome of *P. lanceolata* falls within the size range of Rubiaceae taxa (Yik et al. 2021; Shi et al. 2024; Kang et al. 2026), sharing similar genomic structure, gene count and GC content with its Rubiaceae relatives. Conserved IR genes with minor IR expansion/contraction and identical gene order (Yik et al. 2021; Shi et al. 2024) support their close evolutionary affinity.

In the ML tree, *P. lanceolata* formed a monophyletic clade and exhibited the closest phylogenetic relationship with the tribe Spermacoceae. These results are consistent with those of previous studies (Yik et al. 2021; Shi et al. 2024; Kang et al. 2026), which show close relationships between *P. lanceolata* and most species of the tribe Spermacoceae, as well as broader evolutionary relationships within the Rubiaceae family, despite the historically variable taxonomic treatments of these taxa (Thureborn et al. 2024; Liang et al. 2026).

## Supplementary Material

Supplemental Material

## Figures and Tables

**Figure 1.**
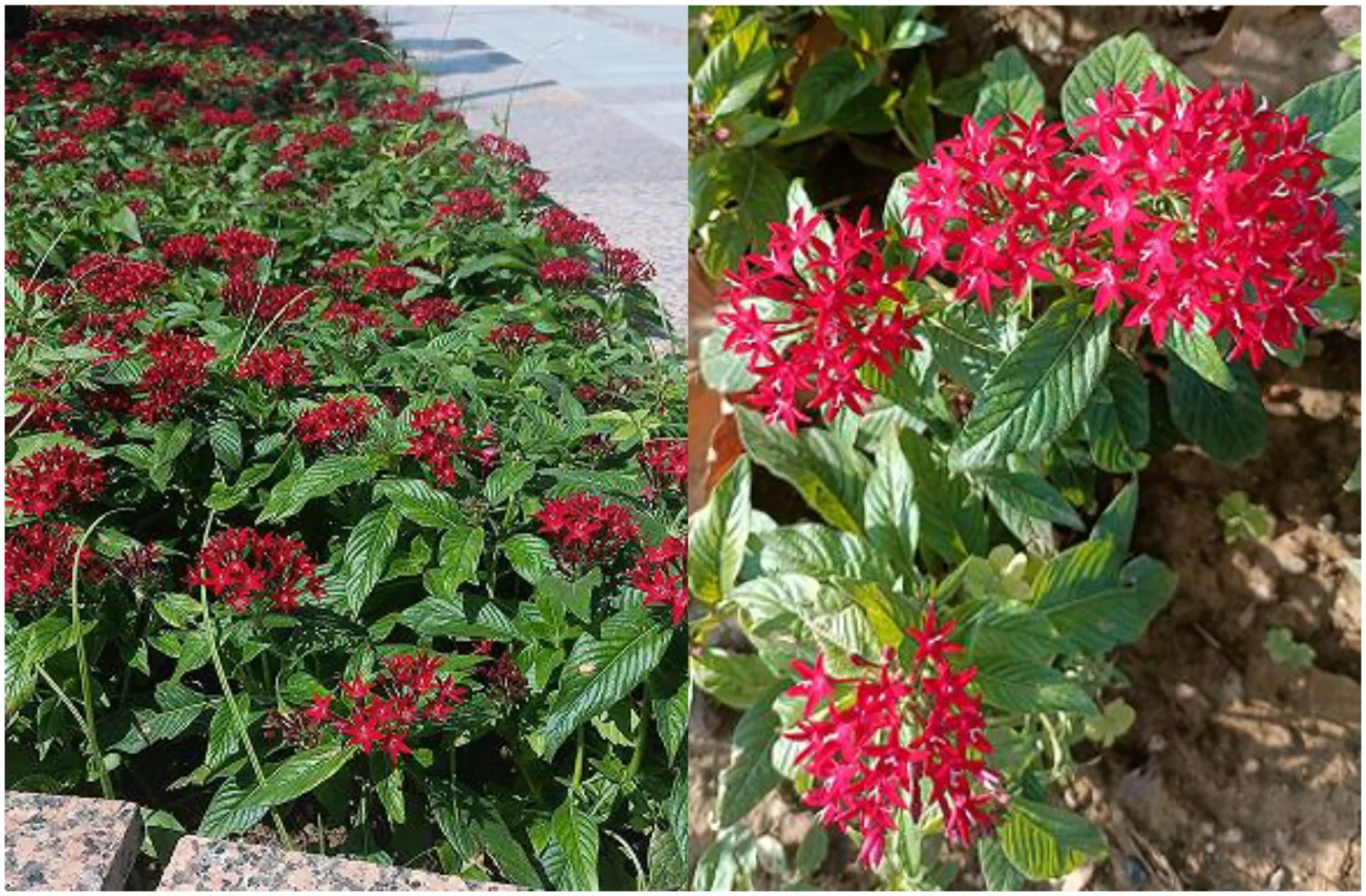
The morphology of *Pentas lanceolata*. Samples of *P. lanceolata* were collected from Wenming East Road, Beicheng Subdistrict, Mudan District, Heze City, Shandong Province, China (35.271538° N, 115.46044° E) and the photographs were taken by Chunfeng Wan. The whorled leaves exhibit fimbriate or setose stipules, and the inflorescences are terminal, dense, corymbose cymes forming showy, hemispherical heads. The flowers are distylous and exhibit a salverform corolla with a long, slender tube and five spreading, star‑shaped lobes.

**Figure 2.**
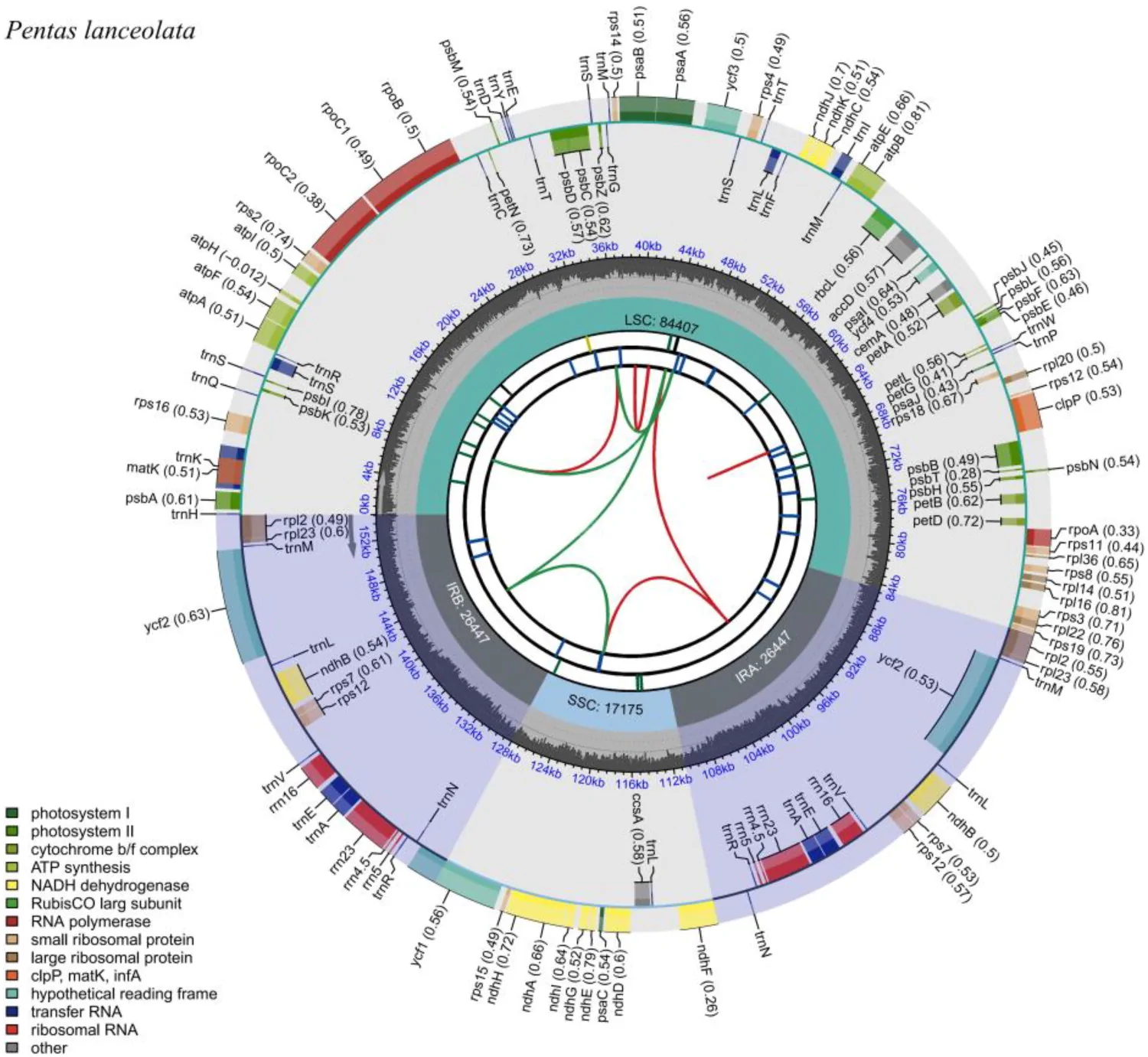
Chloroplast genome map of *Pentas lanceolata*. The map included six tracks. From the center outward, the first track showed dispersed repeats (direct and palindromic repeats), marked by red and green arcs. The second track displayed long tandem repeats, represented as blue bars. The third track showed short tandem repeats or microsatellites, represented as short colored bars. The fourth track included the SSC, IRa, IRb, and LSC regions. The fifth track showed the GC content across the genome. The sixth track presented genes, color-coded by function (see bottom left corner). For protein-coding genes, letters after names indicated subunits or family members; numbers denoted variants. For rRNA genes, the numbers represented the rRNA size in Svedberg units. For tRNA genes, letters indicated recognized amino acids. For unknown-function genes, the numbers referred to hypothetical coding genes. Inner genes were transcribed clockwise; outer genes are transcribed anticlockwise.

**Figure 3.**
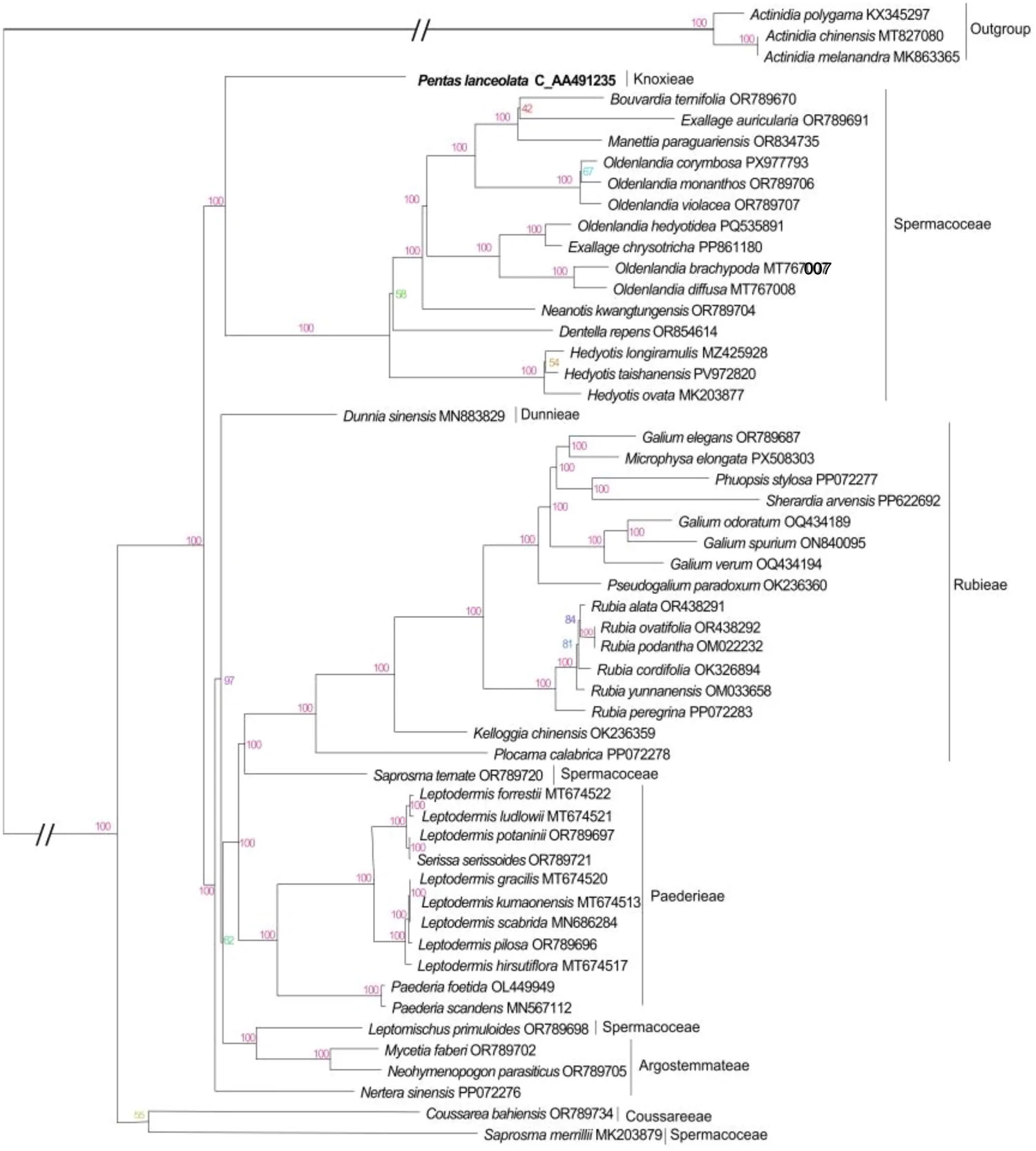
The maximum-likelihood phylogeny of *Pentas lanceolata* and related taxa inferred from 54 concatenated shared chloroplast protein-coding genes (PCGs). Ultrafast bootstrap support values were calculated with 1000 replicates using the ‘-B’ option. Fifty-one species (Rubiaceae, including *P. lanceolata*) and three outgroups (Actinidiaceae) were selected for reconstructing the ML tree. *P. lanceolata* (C_AA491235.1) in this study are labeled in bold font. The three outgroups were *Actinidia polygama* (KX345297) (Wang et al. 2016), *Actinidia chinensis* (MT827080.1), and *Actinidia melanandra* (MK863365.1) (Zhao et al. 2019). The remaining 50 Rubiaceae chloroplast genomes were from *Bouvardia ternifolia* (OR789670.1), *Exallage auricularia* (OR789691.1), *Manettia paraguariensis* (OR834735.1), *Oldenlandia corymbosa* (PX977793.1), *Oldenlandia monanthos* (OR789706.1), *Oldenlandia violacea* (OR789707.1), *Dimetia hedyotidea* (PQ535891.1) (Prasetyo Agung et al. 2026), *Exallage chrysotricha* (PP861180.1), *Oldenlandia brachypoda* (MT767007.1) (Yik et al. 2021), *Oldenlandia diffusa* (MT767008.1) (Yik et al. 2021), *Neanotis kwangtungensis* (OR789704.1), *Dentella repens* (OR854614.1), *Hedyotis longiramulis* (MZ425928.1), *Hedyotis taishanensis* (PV972820.1) (Li et al. 2025), *Hedyotis ovata* (MK203877.1) (Zhang et al. 2019), *Dunnia sinensis* (MN883829.1) (Zhang et al. 2020b), *Galium elegans* (OR789687.1), *Microphysa elongata* (PX508303.1), *Phuopsis stylosa* (PP072277.1), *Sherardia arvensis* (PP622692.1), *Galium odoratum* (OQ434189.1) (Ciborowski et al. 2024), *Galium spurium* (ON840095.1) (Yin et al. 2023), *Galium verum* (OQ434194.1), *Pseudogalium paradoxum* (OK236360.1) (Yu et al. 2025), *Rubia alata* (OR438291.1) (Yu et al. 2025), *Rubia ovatifolia* (OR438292.1) (Yu et al. 2025), *Rubia podantha* (OM022232.1) (Zhao et al. 2023), *Rubia cordifolia* (OK326894.1) (Chen et al. 2023), *Rubia yunnanensis* (OM033658.1) (Zhao et al. 2022), *Rubia peregrina* (PP072283.1), *Kelloggia chinensis* (OK236359.1) (Yang et al. 2022), *Plocama calabrica* (PP072278.1), *Saprosma ternate* (OR789720.1), *Leptodermis forrestii* (MT674522.1) (Zhang et al. 2021), *Leptodermis ludlowii* (MT674521.1) (Zhang et al. 2021), *Leptodermis potaninii* (OR789697.1), *Serissa serissoides* (OR789721.1), *Leptodermis gracilis* (MT674520.1), *Leptodermis kumaonensis* (MT674513.1) (Zhang et al. 2021), *Leptodermis scabrida* (MN686284.1) (Zhang et al. 2020a), *Leptodermis pilosa* (OR789696.1), *Leptodermis hirsutiflora* (MT674517.1) (Yin et al. 2023), *Paederia foetida* (OL449949.1) (Wang et al. 2022), *Paederia scandens* (MN567112.1) (Yin et al. 2023), *Leptomischus primuloides* (OR789698.1), *Mycetia faberi* (OR789702.1), *Neohymenopogon parasiticus* (OR789705.1), *Nertera sinensis* (PP072276.1), *Coussarea bahiensis* (OR789734.1), and *Saprosma merrillii* (MK203879.1) (Zhu et al. 2019).

## Data Availability

The data reported in this paper have been deposited in the GenBase in National Genomics Data Center, Beijing Institute of Genomics, Chinese Academy of Sciences/China National Center for Bioinformation (CNCB-NGDC Members and Partners 2025), under accession number C_AA491235.1 that is publicly accessible at https://ngdc.cncb.ac.cn/genbase/. The associated BioProject, GSA, and BioSample numbers for raw sequencing data in CNCB-NGDC (https://ngdc.cncb.ac.cn/gsa/) were PRJCA055578, CRR3399973, and SAMC8259763, respectively.

## Appendix I

1. Apollo v1.11.6: Berkeley Bioinformatics Open-source Projects (BBOP), Lawrence Berkeley National Laboratory, Berkeley, CA.URL: https://github.com/GMOD/Apollo
2. CPGAVAS2: Center for Bioinformatics, Institute of Medicinal Plant Development, Chinese Academy of Medical Sciences & Peking Union Medical College, Beijing, China.
  URL: http://47.96.249.172:16019/analyzer/home.
3. CPGview: Institute of Medicinal Plant Development, Chinese Academy of Medical Sciences & Peking Union Medical College, Beijing, China.
  URL: http://www.1kmpg.cn/cpgview/
4. CPStools v3.0: School of Traditional Chinese Pharmacy, China Pharmaceutical University, Nanjing, China.
  URL: https://github.com/Xwb7533/CPStools
5. DrawSeqDepth: College of Pharmacy, Heze University, Heze, Shandong, China.
  URL: https://github.com/wlqg1983/DrawSeqDepth
6. FastQC v0.12.1: Babraham Bioinformatics, Babraham Institute, Cambridge, UK.
  URL: https://www.bioinformatics.babraham.ac.uk/projects/fastqc/
7. FigTree v1.4.5: Institute of Evolutionary Biology, University of Edinburgh, Edinburgh, UK.
  URL: http://tree.bio.ed.ac.uk/software/figtree/
8. Gepard v1.40: Technical University of Munich and Helmholtz Zentrum München, Munich, Germany.
  URL: https://github.com/univieCUBE/gepard/raw/master/dist/Gepard-1.40.jar
9. GetOrganelle v1.7.6.1: Kunming Institute of Botany, Chinese Academy of Sciences, Kunming, China.
  URL: https://github.com/Kinggerm/GetOrganelle
10. IQ-TREE v2.0: Computational Phylogenomics Lab, School of Computing, Australian National University, Canberra, ACT, Australia (current maintainer); originally developed at the Center for Integrative Bioinformatics Vienna, University of Vienna & Medical University of Vienna, Vienna, Austria.
  URL: http://www.iqtree.org
11. NovaSeq 6000 platform: Illumina NovaSeq 6000 platform (Illumina, Inc., San Diego, CA); sequencing service provided by Benagen Tech, Wuhan, China.
  URL: https://www.illumina.com/systems/sequencing-platforms/novaseq.html
12. Trimmomatic v0.39: Usadel Lab, Aachen, Germany.
  URL: https://github.com/usadellab/Trimmomatic
