# Supplementary material for: Assembly, annotation, and analysis of the first complete chloroplast genome of medicinal plant Pentas lanceolata (Forssk.) Deflers
Source: Mitochondrial DNA B Resour. 2026 Nov 6;11(12):1389–94. doi: 10.1080/23802359.2026.2753257 (PMC13757842; doi:10.1080/23802359.2026.2753257)
Supplement: Supplementary file 1 [file TMDN_A_2753257_SM4782.docx]

**Table S1. Base composition of the chloroplast genome of *Pentas lanceolata*.**

|  | T (%) | A (%) | C (%) | G (%) | Length (bp) | GC (%) | Proportion (%) |
| --- | --- | --- | --- | --- | --- | --- | --- |
| LSC | 32.67 | 31.54 | 18.38 | 17.40 | 84,407 | 35.79 | 54.64 |
| SSC | 33.61 | 34.18 | 16.95 | 15.25 | 17,175 | 32.21 | 11.12 |
| IRA | 28.88 | 28.23 | 22.21 | 20.69 | 26,447 | 42.89 | 17.12 |
| IRB | 28.23 | 28.88 | 20.69 | 22.21 | 26,447 | 42.89 | 17.12 |
| Total | 31.37 | 30.81 | 19.27 | 18.55 | 154,476 | 37.82 | 100 |
| 1st codon position | 23.91 | 30.48 | 18.88 | 26.73 | 26,188 | 45.61 | - |
| 2nd codon position | 32.58 | 29.20 | 20.17 | 18.05 | 26,188 | 38.22 | - |
| 3rd codon position | 37.68 | 31.54 | 14.28 | 16.49 | 26,188 | 30.77 | - |

**Table S2. List of genes found in the chloroplast genome of *Pentas lanceolata*.**

| Category for genes | Group of genes | Name of genes |
| --- | --- | --- |
| Self-replication | rRNA genes | *rrn16S*(×2)，*rrn23S*(×2)，*rrn4.5S*(×2)，*rrn5S*(×2) |
|  | tRNA genes | *trnH-GUG*, *trnK-UUU*, *trnQ-UUG*, *trnS-GCU*, *trnS-CGA*, *trnR-UCU*, *trnC-GCA*, *trnD-GUC*, *trnY-GUA*, *trnE-UUC(×3)*, *trnT-GGU*, *trnS-UGA*, *trnG-GCC*, *trnM-CAU(×4)*, *trnS-GGA*, *trnT-UGU*, *trnL-UAA*, *trnF-GAA*, *trnI-AAU*, *trnW-CCA*, *trnP-UGG*, *trnL-CAA(×2)*, *trnV-GAC(×2)*, *trnA-UGC(×2)*, *trnR-ACG(×2)*, *trnN-GUU(×2)*, *trnL-UAG* |
|  | Small subunit of ribosome | *rps11*, *rps12(×2)*, *rps14*, *rps15*, *rps16*, *rps18*, *rps19*, *rps*2, *rps*3, *rps*4, *rps*7(×2), *rps*8 |
|  | Large subunit of ribosome | *rpl14*, *rpl16*, *rpl2(×2)*, *rpl20*, *rpl22*, *rpl23 (×2)*, *rpl36* |
|  | DNA dependent RNA polymerase | *rpoA*, *rpoB*, *rpoC1*, *rpoC2* |
| Genes for photosynthesis | Subunits of NADH-dehydrogenase | *ndhA*, *ndhB(×2)*, *ndhC*, *ndhD*, *ndhE*, *ndhF*, *ndhG*, *ndhH*, *ndhI*, *ndhJ*, *ndhK* |
|  | Subunits of photosystem I | *psaA*, *psaB*, *psaC*, *psaI*, *psaJ* |
|  | Subunits of photosystem II | *psbA*, *psbB*, *psbC*, *psbD*, *psbE*, *psbF*, *psbI*, *psbJ*, *psbK*, *psbL*, *psbM*, *psbN*, *psbT*, *psbZ*, *ycf3* |
|  | Subunits of cytochrome  b/f complex | *petA*, *petB*, *petD*, *petG*, *petL*, *petN* |
|  | Subunits of ATP synthase | *atpA*, *atpB*, *atpE*, *atpF*, *atpH*, *atpI* |
|  | Subunit of rubisco | *rbcL* |
| Other genes | Subunit of Acetyl-CoA-carboxylase | *accD* |
|  | c-type cytochrom synthesis gene | *ccsA* |
|  | Envelope membrane | *cemA* |
|  | protein Protease | *clpP* |
|  | Maturase | *matK* |
| Genes of unkown function | Conserved open reading frames | *ycf1*, *ycf2(×2)*, *ycf3*, *ycf4* |

**Table** **S3. Intron-containing genes and their exon/intron lengths in the chloroplast genome of *Pentas lanceolata*.**

| **Gene Name** | **Strand** | **Start** | **End** | **Exon I (bp)** | **Intron I (bp)** | **Exon II (bp)** | **Intron II (bp)** | **Exon III (bp)** |
| --- | --- | --- | --- | --- | --- | --- | --- | --- |
| *trnK-UUU* | - | 1571 | 4135 | 37 | 2492 | 36 |  |  |
| *rps16* | - | 5006 | 6117 | 40 | 845 | 227 |  |  |
| *trnS-CGA* | + | 9408 | 10170 | 32 | 671 | 60 |  |  |
| *atpF* | - | 12121 | 13415 | 145 | 740 | 410 |  |  |
| *rpoC1* | - | 21395 | 24206 | 453 | 742 | 1617 |  |  |
| *ycf3** | - | 43034 | 45050 | 124 | 752 | 230 | 758 | 153 |
| *trnL-UAA* | + | 47529 | 48129 | 35 | 516 | 50 |  |  |
| *trnI-AAU* | - | 51499 | 52162 | 30 | 573 | 61 |  |  |
| *clpP** | - | 69945 | 72078 | 71 | 846 | 294 | 697 | 226 |
| *rpl2* | - | 84427 | 85909 | 391 | 658 | 434 |  |  |
| *ndhB* | - | 94719 | 96946 | 775 | 695 | 758 |  |  |
| *trnE-UUC* | + | 102226 | 103238 | 32 | 941 | 40 |  |  |
| *trnA-UGC* | + | 103302 | 104190 | 37 | 816 | 36 |  |  |
| *ndhA* | - | 120038 | 122289 | 553 | 1160 | 539 |  |  |
| *trnA-UGC* | - | 134694 | 135582 | 37 | 816 | 36 |  |  |
| *trnE-UUC* | - | 135646 | 136658 | 32 | 941 | 40 |  |  |
| *ndhB* | + | 141938 | 144165 | 775 | 695 | 758 |  |  |
| *rpl2* | + | 152975 | 154457 | 391 | 658 | 434 |  |  |

Note: Numbers represent the number of copies. *: indicte two introns.

**A**

**C**

**B**

**E**

**D**

**Figure S1.** **Coverage and sequencing depth of the chloroplast genome of** ***Pentas lanceolata*.** (A) Sequencing depth and coverage map of the complete *P. lanceolata.* chloroplast genome. (B) Sequencing depth and coverage map across the IRa/LSC junction; (C) Sequencing depth and coverage map across the LSC/ IRb junction; (D) Sequencing depth and coverage map across the IRb/SSC junction; (E) Sequencing depth and coverage map across the SSC/IRa junction. The horizontal coordinate was the positional information of the nucleotide acids and the vertical coordinate was the corresponding coverage depth of each base.

**Figure S2.** **Schematic diagram of cis-splicing genes in the chloroplast genome of *Pentas lanceolata*.** Schematic map of the cis-splicing genes in the chloroplast genome. The genes are arranged from top to bottom based on their order on the chloroplast genome. The gene names are shown on the left, and the gene structures are on the right. The exons are shown in black; the introns are shown in white. The arrow indicates the sense direction of the gene.

**Figure S3.** **Schematic diagram of trans-splicing genes (*rps12*) in the chloroplast genome of *Pentas lanceolata*.** The gene has three unique exons. Two of them are duplicated as they are located in the IR regions.

**A**

**B**

**E**

**C**

**D**

**Figure S4.** **Analysis of simple-sequence repeats (SSRs) and long repetitive sequences (LRSs)in the chloroplast genomes of** ***Pentas lanceolata*.** (A) Counts of SSRs categorized by motif type; (B) Regional distribution of SSRs; (C) Abundance of different LSR types; (D) Frequency of LSRs by length class; (E) Distribution of LSRs among different genomic locations.

A total of 20 SSR loci were identified, including 18 mononucleotide repeats (seven A, eight T, and three C), one TA dinucleotide repeat, and one CTTT tetranucleotide repeat (Figure S4A). The SSR motifs were strongly A/T-rich, dominated by A/T mononucleotides and TA dinucleotides. These SSRs were distributed in gene regions (four loci), introns (six loci), and intergenic spacers (ten loci) (Figure S4B).

LSRs were screened using the following criteria: a minimum length of 30 bp, a maximum Hamming distance of three, and ≥90% sequence identity. A total of 20 LSRs were obtained, including six forward, nine palindromic, and five reverse repeats (Figure S4C). By length, seven LSRs were in the 30-31 bp class, five in the 31-38 bp class, and eight were ≥38 bp (Figure S4D). Most LSRs resided in intergenic spacers (14 loci), followed by introns (four loci) and gene regions (two loci) (Figure S4E).

**Figure S5.** **Comparison of LSC, SSC and IR region boundaries in the chloroplast genomes from ten species of Rubiaceae.** The LSC/IRb, IRb/SSC, SSC/IRa, and IRa/LSC junctions are designated as JLB, JSB, JSA, and JLA, respectively.

**Appendix I**

1. **Apollo v1.11.6**: Berkeley Bioinformatics Open-source Projects (BBOP), Lawrence Berkeley National Laboratory, Berkeley, CA, USA.

URL: https://github.com/GMOD/Apollo

2. **CPGAVAS2**: Center for Bioinformatics, Institute of Medicinal Plant Development, Chinese Academy of Medical Sciences & Peking Union Medical College, Beijing, China.

URL: http://47.96.249.172:16019/analyzer/home.

3. **CPGview**: Institute of Medicinal Plant Development, Chinese Academy of Medical Sciences & Peking Union Medical College, Beijing, China.

URL: <http://www.1kmpg.cn/cpgview/>

4. **CPStools v3.0**: School of Traditional Chinese Pharmacy, China Pharmaceutical University, Nanjing, China.

URL: https://github.com/Xwb7533/CPStools

5. **DrawSeqDepth**: College of Pharmacy, Heze University, Heze, Shandong, China. URL: https://github.com/wlqg1983/DrawSeqDepth
6. **FastQC v0.12.1**: Babraham Bioinformatics, Babraham Institute, Cambridge, UK. URL: https://www.bioinformatics.babraham.ac.uk/projects/fastqc/
7. **FigTree v1.4.5**: Institute of Evolutionary Biology, University of Edinburgh, Edinburgh, UK.

URL: <http://tree.bio.ed.ac.uk/software/figtree/>

8. **Gepard v1.40**: Technical University of Munich and Helmholtz Zentrum München, Munich, Germany.

URL: https://github.com/univieCUBE/gepard/raw/master/dist/Gepard-1.40.jar

9. **GetOrganelle v1.7.6.1**: Kunming Institute of Botany, Chinese Academy of Sciences, Kunming, China.

URL: https://github.com/Kinggerm/GetOrganelle

10. **IQ-TREE v2.0**: Computational Phylogenomics Lab, School of Computing, Australian National University, Canberra, ACT, Australia (current maintainer); originally developed at the Center for Integrative Bioinformatics Vienna, University of Vienna & Medical University of Vienna, Vienna, Austria.

URL: <http://www.iqtree.org>

11. **NovaSeq 6000 platform**: Illumina NovaSeq 6000 platform (Illumina, Inc., San Diego, CA, USA); sequencing service provided by Benagen Tech, Wuhan, China.

URL: <https://www.illumina.com/systems/sequencing-platforms/novaseq.html>

12. **Trimmomatic v0.39**: Usadel Lab, Aachen, Germany.

URL: https://github.com/usadellab/Trimmomatic
